# A Compact 2-D photonic crystal biomedical sensor for enhanced glucose concentration detection in urine

**DOI:** 10.1038/s41598-025-87547-x

**Published:** 2025-02-10

**Authors:** Mahmoud M. Hamed, Nazmi A. Mohammed, Kareem A. Badawi

**Affiliations:** 1 Electronics and Communication Engineering Department, Higher Technological Institute, 6Th of October, Giza, Egypt; 2 Electronics and Communication Engineering Department, Al-Madinah Higher Institute for Engineering and Technology, Giza, Egypt; 3 Department of Electronics and Communication Engineering, Giza Engineering Institute, Giza, Egypt

**Keywords:** Cavity, Sensitivity, Quality factor, Detection limit, Compactness, Biophotonics, Assay systems, Kidney diseases, Biomedical engineering

## Abstract

This study introduces a 2-D Photonic Crystal (PhC) biosensor designed, simulated, and evaluated for detecting glucose concentrations in urine by utilizing refractive index variations. The sensor demonstrates exceptional performance, achieving a sensitivity of 20,040.30 nm/RIU for glucose levels ranging from 0–15 mg/dl, a quality factor of 10,424.55, and a detection limit as low as 8 × 10^−10^, surpassing benchmarks reported in the literature. With compact dimensions of 16.8 × 17.6 µm^2^ and compatibility with modern fabrication techniques, the proposed design is well suited for integration into portable diagnostic devices. A comprehensive comparative analysis underscores its superior sensitivity, ultra-high quality factor, and compact design, establishing it as a major advancement in glucose detection technology.

## Introduction

Photonic Crystal (PhC)-based structures have become crucial in Optical Signal Processing (OSP) due to their exceptional advantages, including compact designs, low energy consumption, and high data transfer rates, outperforming conventional platforms like silicon waveguides, semiconductor optical amplifiers, and quantum dot structures ^1–12^. These structures are integral to a wide range of applications, such as resonators^13^, directional couplers^14^, filters^15^, switches^16^, optical modulators^11,17^, oscillators^18^, and sensors^19–21^. As a result, PhC systems are at the forefront of OSP research, highlighting their significant potential for diverse and advanced technological solutions^19^.

The integration of photonic crystal (PhC) technology in sensing applications has gained significant attention due to its ability to enhance sensitivity and quality factors, enabling precise detection through distinct physical properties like reflectance and transmittance^22^. PhC structures have demonstrated their versatility across diverse sensing domains, including high-temperature sensing^23^, pressure sensing^24^, gas sensing^25^, biomedical sensing^26–32^, displacement sensing^33^, liquid sensors^34^, and force-strain sensing^35^. Among these, PhC-based biomedical sensors show exceptional promise, particularly in monitoring glucose levels in blood and urine^36–43^, detecting DNA exposure^44^, identifying cancer cells^45–47^, and diagnosing malaria stages^48,49^. These advanced sensors exemplify the potential of PhC technology in revolutionizing healthcare and all-optical sensing, marking it as a vital focus for future research and development^19–21,26^.

The concentration of glucose in human systems is a critical area of medical research due to its fundamental role as an energy source for physiological functions. Fluctuations in glucose levels significantly affect health, with elevated levels, such as in glucosuria, linked to conditions like diabetes mellitus and its associated complications, including cardiovascular, neuropathic, and retinal issues^50–53^. Monitoring glucose concentration in urine is essential for clinical diagnostics and metabolic studies, as it reflects glycemic control and renal glucose handling. Elevated glucose in urine suggests poor glycemic regulation or renal impairment, while normoglycemia is marked by negligible glucose excretion due to effective renal reabsorption. Accurate urine glucose measurement is vital for diagnosing and managing diabetes, optimizing treatment, and assessing therapeutic outcomes^54–56^.

Modern advancements in biosensor technology, particularly cavity photonic crystal-based designs, have revolutionized urine glucose monitoring by offering real-time, non-invasive, and highly sensitive detection^57,58^. These biosensors exploit the unique optical properties of photonic crystal structures, enabling precise diagnostic capabilities. Key performance parameters such as sensitivity, quality factor, and compactness remain central to biosensor optimization, driving research to address limitations and enhance biomedical sensing technologies^58^. Techniques like integrating photonic crystal waveguides with resonators have significantly improved sensitivity and quality factors, although challenges such as guided-mode resonance limitations and alignment between fabrication and simulation persist^59–61^. These innovations hold promise for advancing diagnostic precision and improving patient outcomes in glucose monitoring systems^57–61^.

This study focuses on developing a glucose concentration detector for urine, operating across a range of concentrations from 0 mg/dl to 10 gm/dl. Utilizing Photonic Crystal (PhC) refractive index measurements, the proposed sensor achieves exceptional accuracy and reliability. The novel design, compatible with modern fabrication technologies, outperforms existing detectors by exhibiting superior sensitivity, quality factor, and compactness. These enhancements establish a robust foundation for the advancement of highly efficient biomedical sensors, demonstrating the potential for future applications in glucose monitoring systems^21,46,47^.

The manuscript is structured to comprehensively address the development of PhC biosensors for glucose detection. Section two highlights the challenges and recent advancements in fabricating these sensors. Section three compares various techniques for detecting glucose concentrations. Section four outlines the proposed design in detail, followed by an evaluation of its performance in Section five. Section six, a comprehensive literature review is conducted, along with a comparison with related PhC based works. Concluding remarks can be found in section seven, followed by a list of the most pertinent references.

### Challenges and advances in fabrication of photonic crystal biosensors for glucose detection

A critical factor in Photonic Crystal (PhC) based sensing is the credibility and reliability of the proposed design. Previous studies have shown that simulation methods such as finite-difference time-domain and plane-wave expansion techniques are effective tools for aligning proposed designs with real-life outcomes. The current study leverages these methodologies, suggesting that the proposed sensor design can achieve high accuracy in detecting varying glucose levels^62–64^.

The primary challenge, however, lies in the fabrication and implementation phases, as ultra-compact structures require extremely high precision and incur significant costs. To mitigate this, the authors adapted a previously fabricated design, adjusting its parameters for better alignment with the objectives of this study. Nevertheless, the high costs associated with reliable fabrication remain a barrier to immediate realization^65^.

Fabrication techniques such as Electron-Beam Lithography (EBL) provide the resolution required for intricate PhC designs critical for high sensitivity but are costly and not suited for large-scale production. Nanoimprint Lithography (NIL) and Laser Interference Lithography (LIL) offer more scalable and affordable alternatives but may lack the precision necessary for ultra-sensitive glucose detection. Emerging methods like soft lithography and 3D printing provide flexibility for portable or wearable applications, though these often compromise on resolution and reliability^66,67^.

Ultimately, the trade-off between cost, scalability, and precision determines the choice of fabrication method for PhC-based glucose sensors. A promising approach is to enhance simulation accuracy and employ innovative, scalable fabrication techniques to achieve affordability^65^.

### Comparative between different techniques for glucose concentration detection

A variety of techniques are employed to measure glucose concentrations in urine, ranging from traditional chemical methods like test strips and enzymatic techniques to advanced technologies such as biosensors, near-infrared spectroscopy, and microfluidic devices. These methods are widely used for diagnosing and monitoring diabetes, each offering distinct benefits and limitations. This diversity allows for tailored approaches depending on accuracy, cost, and application requirements^57,68–76^.

Photonic crystal sensors offer distinct advantages over traditional and modern glucose detection methods. By utilizing periodic nanostructures that interact with light, these sensors are highly sensitive to changes in glucose concentration^36,37^. They provide real-time monitoring, allowing for instant glucose readings without the need for time-consuming laboratory analysis. This capability enhances diabetes management by enabling quicker responses to fluctuations in glucose levels, promoting more proactive care^71^.

Photonic crystal sensors offer high sensitivity and accuracy in glucose concentration detection, capable of detecting even subtle variations within the normal range^36–38^. This precision is critical for reliable diabetes management. However, these sensors face challenges due to their high initial cost and the complexity of their fabrication, which involves advanced manufacturing techniques^57,72,75^. As a result, their production expenses are higher than those of traditional glucose monitoring methods or other sensor types. The benefits, drawbacks, and costs of various glucose measurement methods are summarized in Table 1^36–38,57,72–76^.

**Table 1:** Pros, cons and cost of some glucose concentration in urine measurement Approaches.

Various approaches for measuring glucose concentrations in urine	Advantages	Disadvantages	Cost
Chemical Test Strips	Simple, inexpensive, and easy to use. No specialized equipment is required	Semi-quantitative results, not very accurate or sensitive. Can be influenced by factors like hydration status and medications	Test strips available over the counter at pharmacies or online retailers can range in price. Typically, you can find packages containing 25 to 100 strips. The cost per strip can vary from approximately $0.15 to $0.50 or more, depending on the brand and the quantity purchased
Enzymatic Techniques	More accurate and specific than dipstick tests. Can provide quantitative results. Enzymes can be tailored for specificity	Still subject to interference from substances other than glucose. Requires careful handling and reagent storage	Enzymatic test strips or reagent kits designed for urine glucose measurement are available for over-the-counter use. These kits often include multiple strips or reagents. The cost per strip or test can range from approximately $0.10 to $0.50 or more, depending on the brand, quantity, and features of the kit
Glucose Meters	Provides rapid results, especially useful for self-monitoring by individuals with diabetes. Some models are portable and easy to use	Requires test strips and regular calibration. Accuracy can be affected by factors such as temperature and hematocrit levels. Blood contamination can affect readings	Some glucose meters come with more advanced features, such as data storage, Bluetooth connectivity, and compatibility with smartphone apps. These meters may be priced at the higher end of the range, typically ranging from $50 to $100 or more
Biosensors	Offers real-time monitoring, high sensitivity, and specificity. Can be integrated into wearable devices for continuous monitoring	Initial cost can be higher. Requires calibration and maintenance. Sensitivity can decrease over time due to enzyme degradation	Biosensors used in laboratory or research settings can vary widely in price, from a few hundred dollars to several thousand dollars or more, depending on the complexity and features of the biosensor
Laboratory-Based Procedures	High accuracy, precision, and reliability. Can detect and quantify multiple analytes simultaneously	Expensive and requires specialized equipment and trained personnel. Longer turnaround time compared to point-of-care methods	In-House Laboratory Testing: If a laboratory has the necessary equipment and expertise to conduct glucose concentration measurements in urine in-house, the cost may primarily involve reagents, consumables, and labor The cost can range from a few dollars to several dollars per test, depending on the specific method and reagents used External Laboratory Testing: Many healthcare facilities send urine samples to external clinical laboratories for testing. In this case, the cost can include fees for sample collection, transportation, and analysis The cost can vary widely depending on the laboratory and geographic location. It may range from $10 to $50 or more per test
Colorimetric Assays	Simple and cost-effective. Can be adapted for high-throughput testing	Semi-quantitative and less accurate compared to some other methods. Color interpretation can be subjective	Laboratory-grade colorimetric assay kits designed for research or clinical use can vary in price. The cost of these kits may range from a few dollars to several dollars or more per test, depending on the brand and the specific features of the kit
Near-Infrared Spectroscopy	Non-invasive, fast, and can be used for multiple analytes. Can provide quantitative results without reagents	Requires specialized equipment and expertise. Calibration is necessary for accurate results	Portable Near-Infrared Spectroscopy devices, which may have more limited capabilities but offer convenience and mobility, can be more affordable. Prices for these devices can start from a few thousand dollars and may range up to several thousand dollars
Microfluidic Devices	Small sample volumes required. Enables rapid testing and integration with other diagnostic functions	Requires precise fabrication. Initial development costs can be high. Sensitivity might be affected by the miniaturization process	Some microfluidic devices may be custom-designed or specialized for particular research or clinical applications. The cost of these customized devices can vary widely based on complexity and design requirements
Photonic crystal	Photonic crystal sensors generally offer higher sensitivity and specificity, making them suitable for detecting low concentrations of analytes like glucose and provide real-time data, which can be valuable for studying kinetic interactions	Photonic crystal methods can be more complex to implement due to fabrication and equipment requirements. They can also be more expensive	As the photonic crystal technology transitions into mass production, it is anticipated that the cost of photonic crystal sensors will significantly decrease from several hundred dollars to just a few dollars. This reduction in cost, combined with the inherent benefits of photonic crystal sensors such as enhanced sensitivity, quality, and compactness, positions them as promising future sensing techniques. Furthermore, their compact design allows for portability, making photonic crystal sensors adaptable for use in various locations

The proposed PhC biosensor stands out for its compact design, high sensitivity, and specificity without the need for reagents or calibration^36–38^. Its cost-effectiveness is expected to improve with mass production, making it ideal for non-invasive, real-time glucose monitoring^57,72^. While traditional methods like chemical strips are inexpensive but less precise, and advanced technologies like spectroscopy are costly, the PhC biosensor combines high precision, portability, and affordability^57,72,76^. Although initially expensive, it is projected to become more affordable, offering a cost-effective solution for diverse diagnostic applications^57^.

## Methods

### The proposed design

As previously mentioned, this design can be constructed utilizing modern technology, with its foundation derived from^77^. Importantly, it should be emphasized that this fabricable design can detect various levels of glucose concentration in urine, as demonstrated in the upcoming section. Moreover, this section will introduce evaluation parameters for the sensing process, namely sensitivity, quality factor, and compactness.

**Fig. 1:**
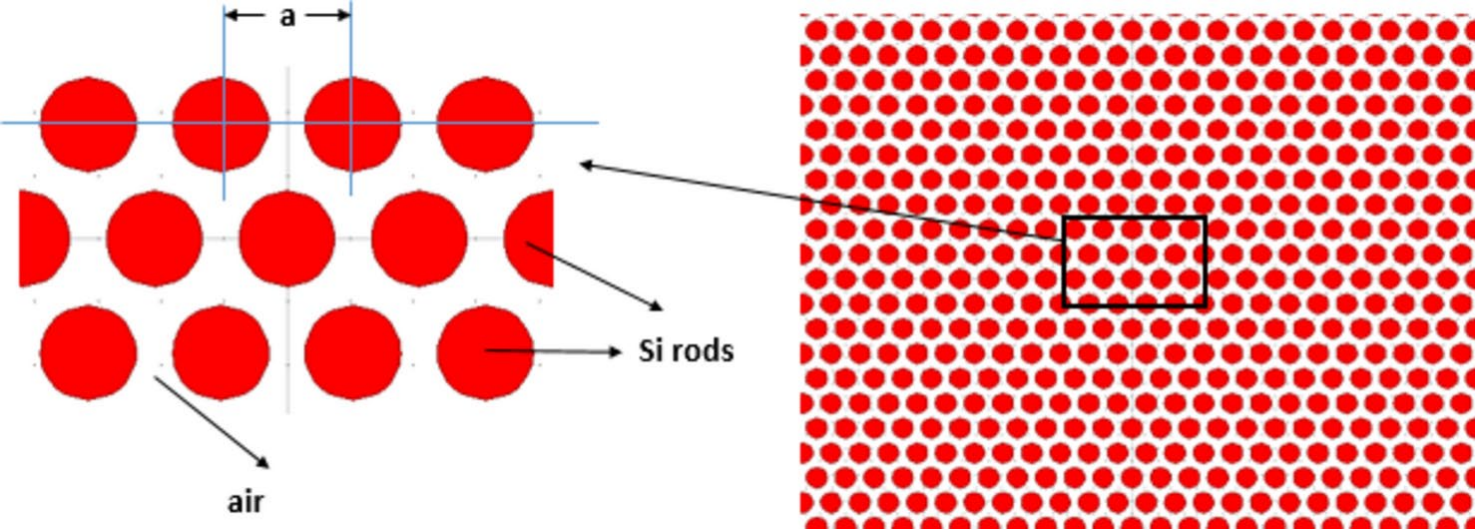
Depicts the suggested configuration for the initial design without defects.

**Fig. 2:**
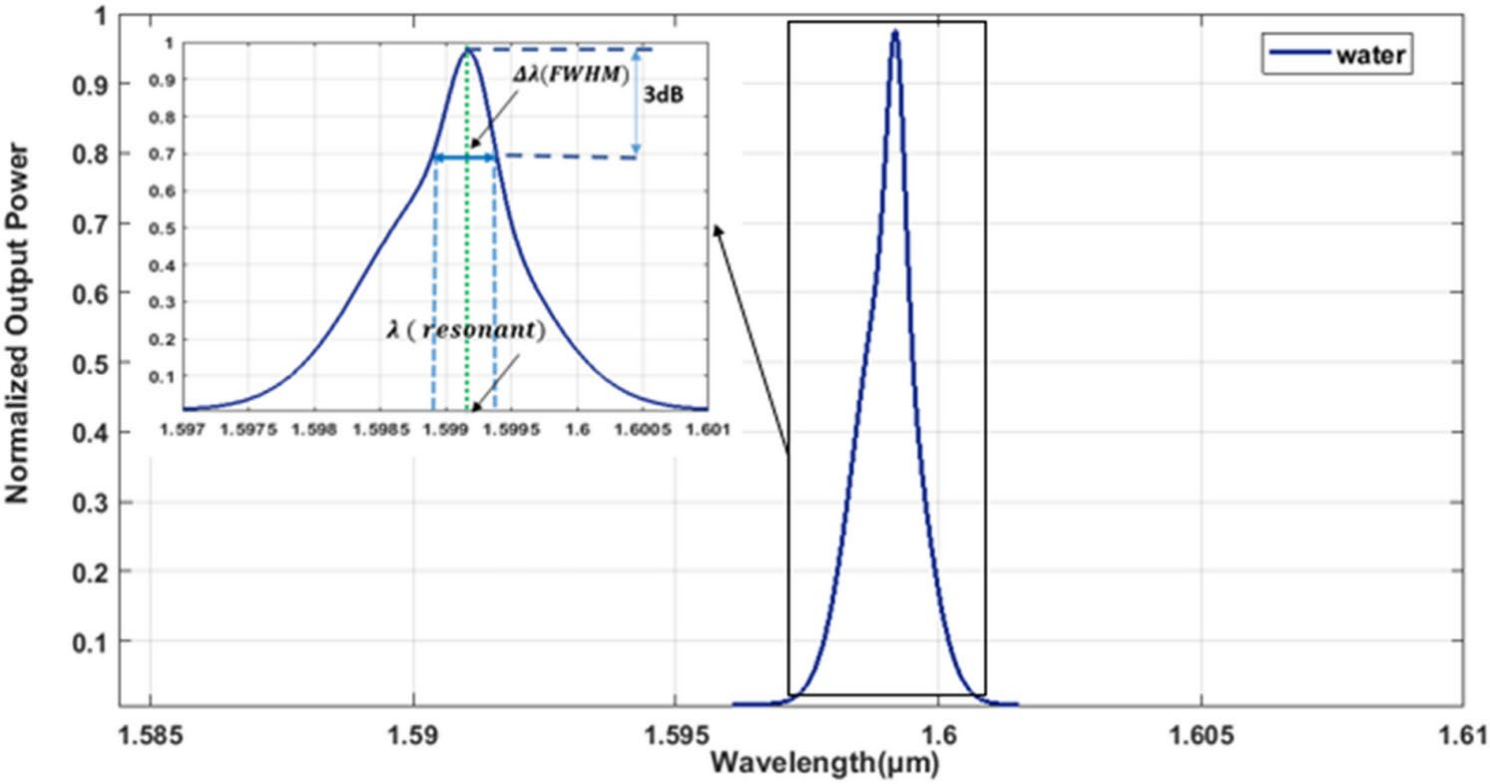
Normalized transmission spectra of the provided configuration, presuming that the substance inside the cavities is normal analyte (i.e. water).

Figure 1 displays the configuration of the initial design, employing a 2D hexagonal lattice composed of Silicon (Si) rods. The radius of these Si rods measures r = 0.3a, where "a" denotes the lattice constant, representing the distance between the centers of the two rods, set at 880 nm. The range of frequencies where the light beam cannot propagate is referred to as the photonic band gap^78^. To compute this photonic band gap, the PWE (Plane Wave Expansion) method is utilized^79^. Figure 2 illustrates the transmission spectrum corresponding to the suggested configuration, assuming that the analyte within the cavities is in its standard state (i.e., containing water). The resonant wavelength, depicted in Fig. 2, is measured at 1.599 μm.

**Fig. 3:**
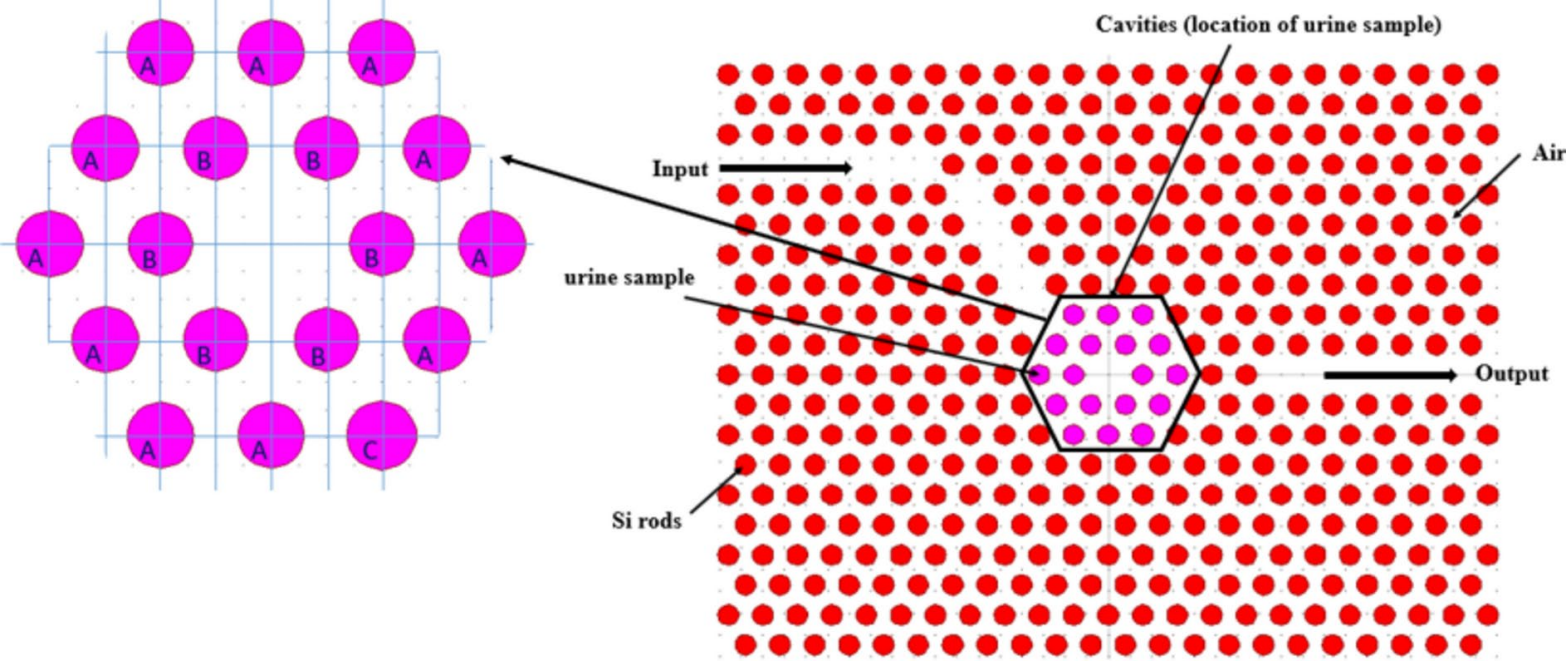
The concluding phase of the initial proposed design incorporating defects.

Figure 3 illustrates the proposed design incorporating essential defects crucial for achieving high-performance glucose concentration detection in urine. As mentioned earlier, the design aims to meet specific targets, including ease of fabrication, exceptional sensitivity with reasonable quality factors, and compactness. These objectives are accomplished through the optimization of two parameters. Firstly, by modifying the radii of the cavities labeled A, B and C, as depicted in Fig. 3. Secondly, by strategically positioning these cavities within the structure of the proposed design, which will be discussed in the subsequent section. Ultimately, the proposed structure for glucose concentration detection in urine utilizes the resonant wavelength as the measuring indicator, exhibiting variations in response to changes in the index of refraction of the cavities.

The quality factor is calculated through a specific method:

1 $$Q= \frac{\lambda ( resonant)}{\Delta \lambda (FWHM)}$$

whereas, λ (resonant) represents the wavelength at which resonance occurs, and Δλ (FWHM) denotes the wavelength difference obtained at the Full Width at Half Maximum (FWHM) point.

The sensitivity (S) is calculated through a specific method:

2 $$S= \frac{\Delta \lambda }{\Delta n} \left(nm.{RIU}^{-1}\right)$$

whereas, $\Delta \lambda$ refers to the change in resonant wavelength, and Δn represents the change in refractive index.

The Detection Limit (DL) is necessary, which indicates the minimum detectable change in the refractive index unit (RIU) that the sensor is capable of identifying. The DL can be formulated as follows:

3 $$DL= \frac{{\lambda }_{o} }{10QS} (RIU)$$

whereas, ${\lambda }_{o}$ is the resonant wavelength. Q and S are the quality factor and sensitivity respectively^80,81^.

**Fig. 4:**
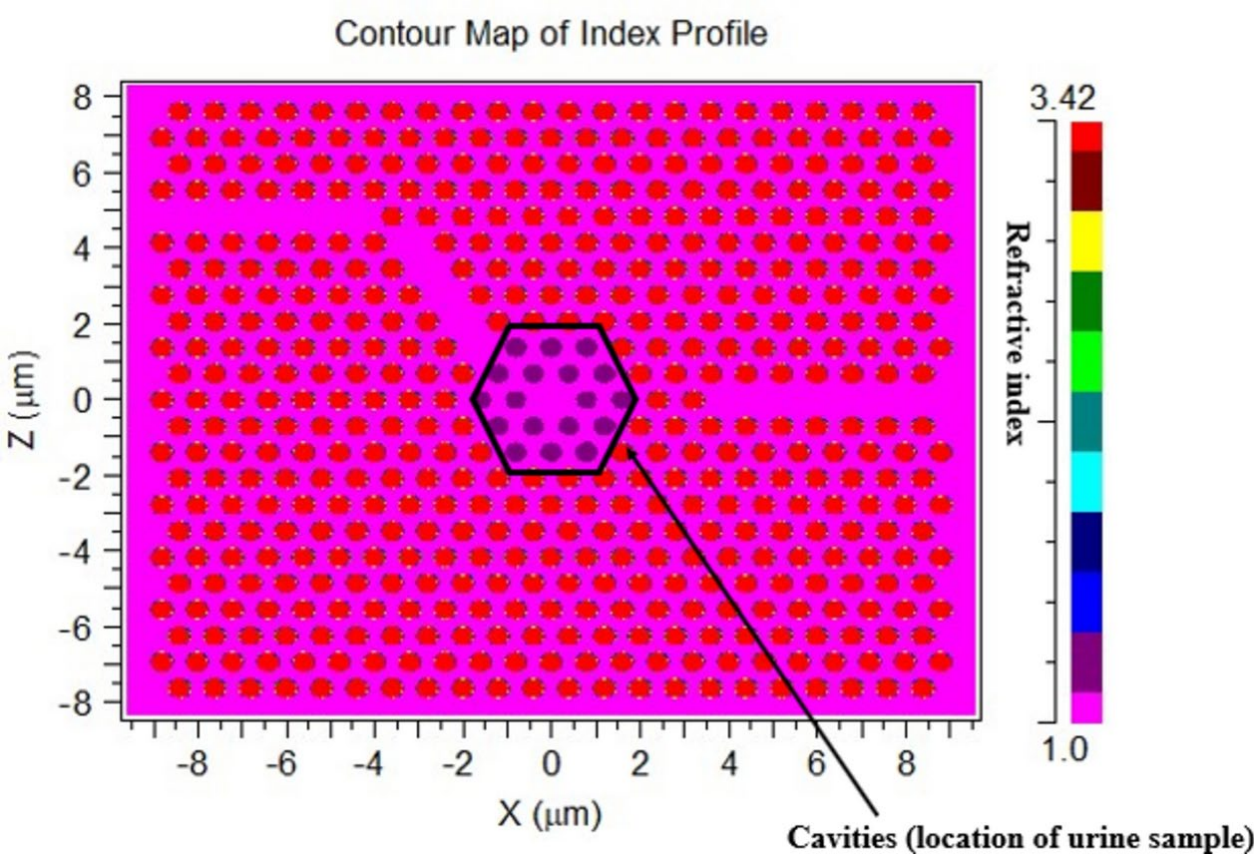
The contour map of the index profile for the proposed design.

Figure 4 presents a contour map of the index profile visually represents the spatial distribution of refractive index variations within the proposed design. It offers a clear and concise depiction of how the refractive index changes across the structure, aiding in the understanding and optimization of light interaction.

## Result and discussion

The presented design is executed and simulated through the implementation of the Finite-Difference Time-Domain (FDTD) technique. This method is harnessed for numerical simulations to accurately solve Maxwell’s equations, which effectively describe a wide range of photonic structures and applications^82^.

**Fig. 5:**
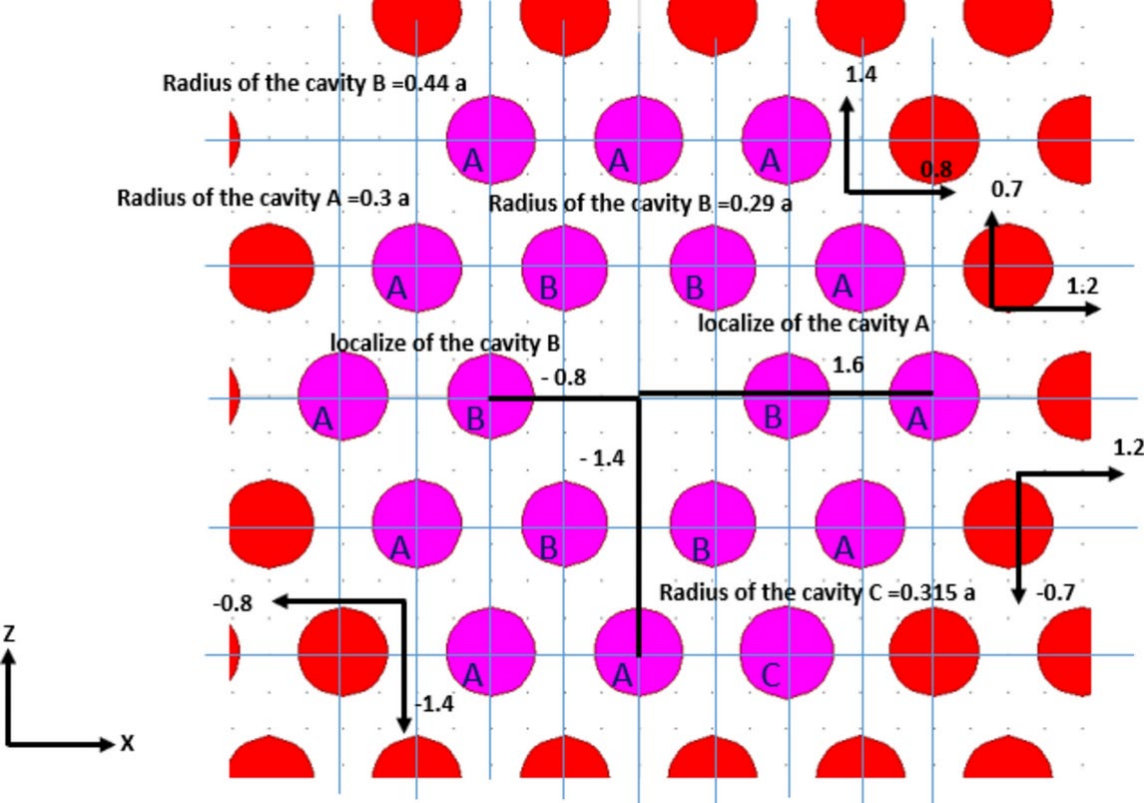
The optimal values of cavity radius and shift, essential for accomplishing the objectives of the proposed design.

During the design stage, an extensive optimization process was performed on the silicon rods labeled A, B, and C, which constitute the proposed cavity as illustrated in Fig. 3. This process involved fine-tuning the radius of each rod and their positions relative to the lattice origin. The initial parameter values are outlined in Table 2, aimed at achieving the design objectives of extremely high sensitivity, ultra-high quality factor, and compactness. As shown in Table 2, ten iterative steps were conducted to determine the optimal conditions, with the results at step 10 corresponding to the goals of the proposed design. Figure 5 illustrates the values that attain the highest sensitivity in glucose concentration detectors in urine and demonstrate an acceptable quality factor, surpassing those reported in the relevant literature.

**Table 2:** Optimization process for the radius of Si rods A, B and C with corresponding resonant wavelength, sensitivity and quality factor for the design stage at 10 gm/dl of glucose concentration in urine.

Step number	Radius of Cavity A ( x a )	Radius of Cavity B ( x a )	Radius of Cavity C ( x a )	Resonant wavelength (µm)	Sensitivity (nm/RIU)	Quality factor ( unit less)
1	0.3	0.44	0.3	1.7010	5870.59	8602.1
2	0.3	0.35	0.3	1.7030	6076.47	8250
3	0.3	0.29	0.29	1.7005	5970.59	8502.5
4	0.29	0.29	0.31	1.6996	5917.65	8498
5	0.32	0.28	0.31	1.7021	6100.59	8611.1
6	0.3	0.35	0.315	1.7031	6176.47	8521.15
7	0.32	0.285	0.315	1.7020	6073.53	5674.17
8	0.315	0.29	0.315	1.70215	5870.59	7511.1
9	0.31	0.3	0.315	1.7022	6070.59	8511
10	0.3	0.29	0.315	1.7018	6051.76	10,636.75

**Fig. 6:**
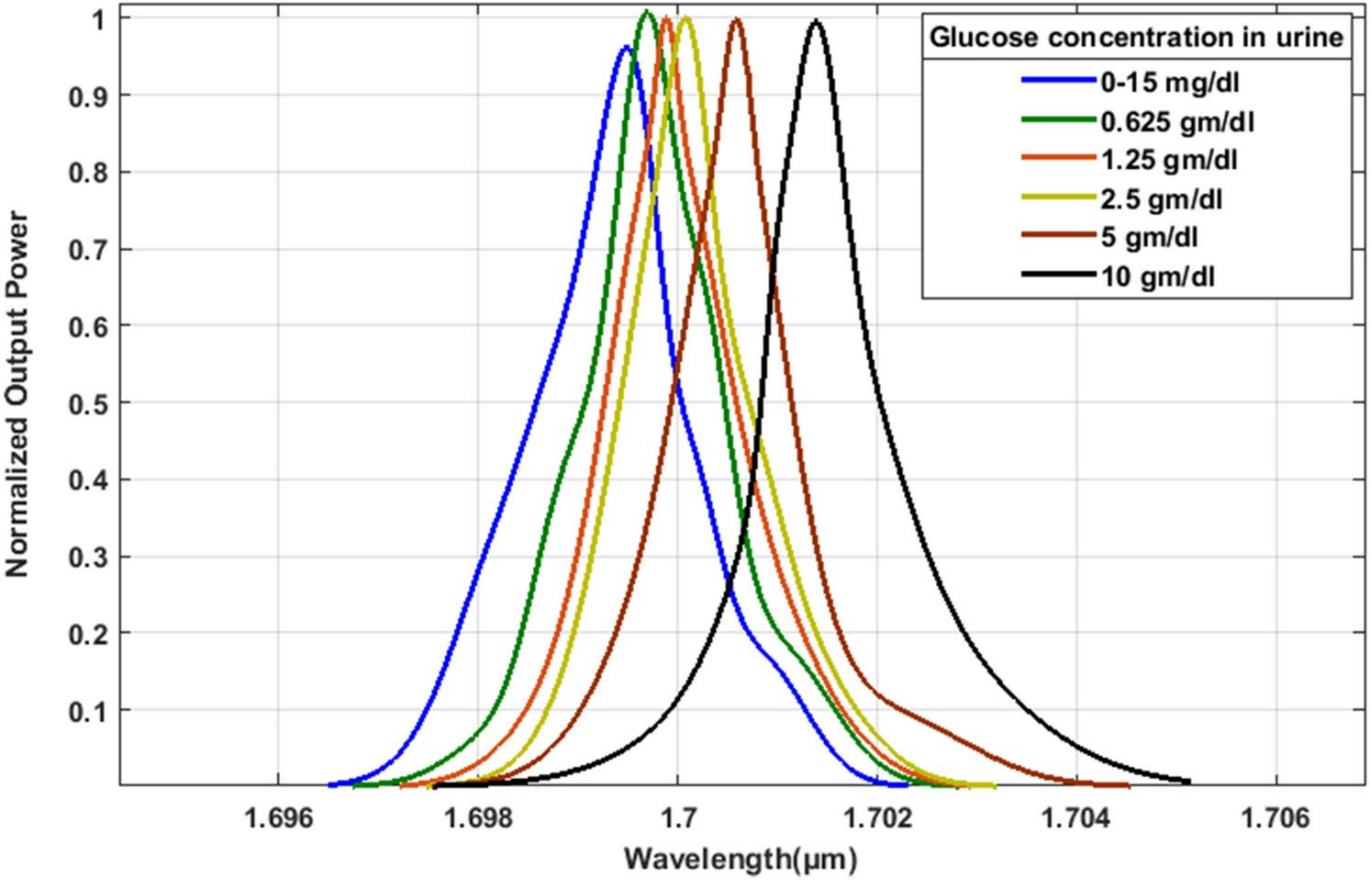
Normalized transmission spectra of the presented design with optimized parameters.

Figure 6 illustrates the transmission spectra output for the optimized design across various glucose concentrations in urine. In Table 3, you can find the corresponding data for the refractive index, resonant wavelength, sensitivity, quality factor, detection limit, and compactness.

**Table 3:** Resonant wavelength, sensitivity, and quality factor of glucose concentration in urine for the presented design.

Different glucose concentration in urine	Refractive index	Resonant wavelength (µm)	Sensitivity (nm/RIU)	Quality factor (unit less)	Detection Limit (RIU)	Size (µm^2^)
0 – 15 mg/dl	1.335	1.6992	20,040.30	10,424.55	0.0000000008	16.8 × 17.6
0.625 gm/dl	1.336	1.6995	16,765.67	10,116.63	0.0000000010	
1.25 gm/dl	1.337	1.6998	14,404.29	10,623.94	0.0000000011	
2.5 gm/dl	1.338	1.7000	12,633.50	10,897.87	0.0000000012	
5 gm/dl	1.341	1.7009	9265.91	11,339.50	0.0000000016	
10 gm/dl	1.347	1.7018	6051.76	10,636.75	0.0000000026	

In conclusion, this design achieves remarkable sensitivity and quality factors. As demonstrated in section 4, these levels surpass those of related glucose concentration in urine sensors using different techniques. However, if ultra-quality factors, ultra sensitivity and exceptional detection limit are the specific objectives of the sensor’s operation, they can be achieved through the proposed design.

## Literature review

As noted in Sect.  1, a comprehensive comparison between the design introduced in this study and those found in prior relevant literature concerning glucose concentration detection in urine will be thoroughly outlined in this section. This comparison aims to provide a detailed overview of the advancements in glucose concentration detection in urine using various techniques and structures based on photonic crystals, while also affirming the novelty of the proposed design.

The presented sensor demonstrates sensitivity, quality factor, and detection limit that surpass the corresponding benchmarks for glucose concentration detection in urine. The proposed design exhibits exceptional sensitivity, achieves remarkable quality factor performance, and excels in detection limit across various levels of glucose concentration in urine. Additionally, an investigation and comparison are provided in Table 4.

**Table 4:** Comparative Analysis between Literature on Glucose Concentration Sensors and the Present Study.

Ref	Year	Target Of sensing	Various glucose concentration biomarkers	Sensitivity	Quality Factor ( unit less)	Detection limit( RIU)	Operating Technique	Size (µm^2^ )
^36^	2017	Glucose in blood	165 -180 mg/dl	NA^1^	264	NA^1^	ring resonator	21 × 21
			182 mg/dl	NA^1^	260	NA^1^		
			205 -289 mg/dl	NA^1^	252	NA^1^		
			342 mg/dl	NA^1^	269	NA^1^		
		Glucose in urine	0–15 mg/dl	NA^1^	264	NA^1^		
			0.625 gm/dl	NA^1^	269	NA^1^		
			1.25 gm/dl	NA^1^	260	NA^1^		
			2.25 gm /dl	NA^1^	252	NA^1^		
			5 gm / dl	NA^1^	217	NA^1^		
^37^	2020	Glucose in urine sensor 1	0–15 mg/dl	500 nm/RIU	3094	NA^1^	cavity	14.93 × 8.8
			0.625 gm/dl	500 nm/RIU	3860	NA^1^		
			1.25 gm/dl	500 nm/RIU	3685	NA^1^		
			2.25 gm /dl	500 nm/RIU	3871	NA^1^		
			5 gm / dl	470 nm/RIU	3228	NA^1^		
			10 gm / dl	420 nm/RIU	3044	NA^1^		
		Glucose in urine sensor 2	0–15 mg/dl	400 nm/RIU	5540	NA^1^	cavity	15.7 × 9.47
			0.625 gm/dl	300 nm/RIU	5180	NA^1^		
			1.25 gm/dl	400 nm/RIU	5175	NA^1^		
			2.25 gm /dl	500 nm/RIU	5545	NA^1^		
			5 gm / dl	366 nm/RIU	4980	NA^1^		
			10 gm / dl	416 nm/RIU	4870	NA^1^		
^38^	2015	Glucose in urine	0–15 mg/dl	NA^1^	NA^1^	NA^1^	line defect	NA^1^
			0.625 gm/dl	NA^1^	NA^1^	NA^1^		
			1.25 gm/dl	NA^1^	NA^1^	NA^1^		
			2.25 gm /dl	NA^1^	NA^1^	NA^1^		
			5 gm / dl	NA^1^	NA^1^	NA^1^		
			10 gm / dl	NA^1^	NA^1^	NA^1^		
		Glucose in Blood	165 -180 mg/dl	NA^1^	NA^1^	NA^1^		
			182 mg/dl	NA^1^	NA^1^	NA^1^		
			205 mg/dl	NA^1^	NA^1^	NA^1^		
			220 mg/dl	NA^1^	NA^1^	NA^1^		
			240 mg/dl	NA^1^	NA^1^	NA^1^		
			255 mg/dl	NA^1^	NA^1^	NA^1^		
			289 mg/dl	NA^1^	NA^1^	NA^1^		
			342 mg/dl	NA^1^	NA^1^	NA^1^		
^39^	2017	Glucose in Blood	30 g/l	333.3 nm/RIU	171.7	NA^1^	rhombic ring resonator	11.4 × 9.8
			90 g/l	666.6 nm/RIU	110.5	NA^1^		
			150 g/l	1000 nm/RIU	155.1	NA^1^		
			210 g/l	666.6 nm/RIU	178.5	NA^1^		
			270 g/l	666.6 nm/RIU	153.9	NA^1^		
			330 g/l	1000 nm/RIU	173.1	NA^1^		
^40^	2020	Glucose in Blood	5g/l	546.72 nm/RIU	2066.24	1.44 × 10^–4^	Capsule cavity	NA^1^
			10 g/l	529.9 nm/RIU	2069.26	1.48 × 10^–4^		
			15 g/l	524.29 nm/RIU	2069.65	1.5 × 10^–4^		
			20 g/l	521.49 nm/RIU	2070.04	1.51 × 10^–4^		
			25 g/l	519.13 nm/RIU	2075.71	1.51 × 10^–4^		
			30 g/l	516.72 nm/RIU	2078.75	1.51 × 10^–4^		
			35 g/l	516.44 nm/RIU	2084.47	1.51 × 10^–4^		
^41^	2020	Glucose in Blood	15 g/l	624.7904 nm/RIU	30,418.329	NA^1^	resonant microcavity	NA^1^
			30 g/l	617.4839 nm/RIU	31,050.726	NA^1^		
			45 g/l	623.5287 nm/RIU	33,324.337	NA^1^		
			60 g/l	621.2297 nm/RIU	34,505.712	NA^1^		
			75 g/l	620.0218 nm/RIU	32,135.634	NA^1^		
			90 g/l	619.1979 nm/RIU	33,022.014	NA^1^		
^42^	2023	Glucose in Blood	165 -180 mg/dl	–-	310	NA^1^	ring resonator	NA^1^
			182 mg/dl	400 nm/RIU	292	NA^1^		
			205 -289 mg/dl	396.7 nm/RIU	286	NA^1^		
			342 mg/dl	390 nm/RIU	284	NA^1^		
		Glucose in urine	0–15 mg/dl	–-	310	NA^1^		
			0.625 gm/dl	400 nm/RIU	298	NA^1^		
			1.25 gm/dl	396.7 nm/RIU	292	NA^1^		
			2.25 gm /dl	390 nm/RIU	286	NA^1^		
			5 gm / dl	391.7 nm/RIU	274	NA^1^		
This work	2024	Glucose in urine	0–15 mg/dl	20,040.30 nm/RIU	10,424.55	8 × 10^–10^	cavity	16.8 × 17.6
			0.625 gm/dl	16,765.67 nm/RIU	10,116.63	10 × 10^–10^		
			1.25 gm/dl	14,404.29 nm/RIU	10,623.94	11 × 10^–10^		
			2.25 gm /dl	12,633.50 nm/RIU	10,897.87	12 × 10^–10^		
			5 gm / dl	9265.91 nm/RIU	11,339.50	16 × 10^–10^		
			10 gm / dl	6051.76 nm/RIU	10,636.75	26 × 10^–10^		

^1^NA is Not Available.

## Conclusion

This work represents a significant step towards the advancement of future PhC biosensors. The introduced design, which can be feasibly realized using contemporary technology, achieves notable sensitivity, an ultra-high-quality factor, and a remarkable detection limit compared to relevant studies. Notably, this design is the first of its kind to operate within the spectrum of glucose concentrations in urine. The sensor achieves sensitivity ranging from 20,040.30 nm/RIU (0–15 mg/dl) to 6051.76 nm/RIU (10 gm/dl), with corresponding quality factors from 10,424.55 to 10,636.75. The detection limit reaches 8 × 10^−10^, establishing a new standard in glucose sensing. The proposed device, with a compact size of 16.8 × 17.6 µm^2^, is compatible with current fabrication methods, making it viable for practical applications. A comprehensive comparison with existing literature on glucose concentration detection in urine is conducted, reinforcing the extraordinary levels of performance indicated above.

To implement this design in practical applications, the fabrication process must employ advanced lithographic techniques to precisely construct the photonic crystal structures, ensuring high accuracy and repeatability. Subsequently, the sensor should undergo rigorous testing in real-world conditions, followed by optimization for scalability, cost-efficiency, and integration into wearable or portable diagnostic devices. Future research should focus on enhancing its ability to detect multiple analytes simultaneously, broadening its applicability in comprehensive diagnostics and health monitoring, and establishing it as a versatile and cost-effective solution in medical technology.

## Data Availability

The authors confirm that the data supporting the findings of this study are available within the article.
